# Elevated Risk of Infections after Spinal Cord Surgery in Relation to Preoperative Pressure Ulcers: a Follow-up Study

**DOI:** 10.1038/s41598-018-32157-z

**Published:** 2018-09-19

**Authors:** Lei-luo Yang, Wu-xun Peng, Chun-qing Wang, Qing Li

**Affiliations:** Department of Orthopaedics, The Affiliated Hospital of Guizhou Medical University, NO 28 Guiyi Street, Guiyang, Guizhou 550001 China

## Abstract

Factors associated with infections after spinal cord surgery were not fully understood. This study aimed to evaluate whether preoperative pressure ulcers was a risk factor of infections after spinal cord operation. A 1:1 matched follow-up study was performed in a tertiary referral center in southwest China between 2010 and 2015. Risk ratios (RRs) and 95% confidence intervals (CIs) were calculated using unconditional logistic regression analysis. A total of 334 patients with spinal cord surgery were recruited (167 patients with preoperative pressure ulcers and 167 patients without preoperative pressure ulcers). Participants previously exposed to pressure ulcers had an elevated risk of infections post spinal cord operation including surgical site infection (RR: 2.3, 95% CI: 1.1, 4.7), pneumonia (RR: 2.4, 95% CI: 1.1,5.3), urinary tract infection (RR: 2.8, 95% CI: 1.1, 7.3), any kinds of postoperative infections (RR: 3.4, 95% CI: 2.1, 5.6) and 30-day postoperative hospitalization for infections (RR: 2.6, 95% CI: 1.1, 6.0). The associations between preoperative pressure ulcers in stage III to IV and postoperative infections were also pronounced, but towards null in stage I to II. The study showed an increased risk of infections after spinal cord surgery in patients with preoperative pressure ulcers, indicative of an urgent need for monitoring postoperative infections and medical treatment for patients with pressure sores.

## Introduction

Spinal cord injury (SCI) remains a devastatingly neurologic disease worldwide^1,2^. Factors affecting infections after spinal cord surgery might include old age, operation time, preoperative infection, malnutrition and etc.^3^. Infections after spinal cord surgery may increase hospital stay length, mortality rates and poor quality of life, which added health-care costs and financial burden to families and societies^4^. Due to antibiotic resistant pathogens in hospital-associated infections, it highlights the importance of preventing risk factors of infection in patients with spinal cord operation^5^.

Pressure ulcer is a set of complications which are featured with damage and defect of skin tissue^4^. In United States, there were 1.3–3 million patients suffering from pressure ulcer in 2003 and 2013^6,7^. Patients with pressure sores were considered to have an elevated risk of inflammation, infection and mortality^8,9^. Recently, a retrospective cohort study revealed that patients with preoperative pressure sores were considered to have an increased risk of adverse outcomes (including postoperative infection) after major surgery (including spinal cord operation)^10^, indicative of a potential risk factor to infections after spinal cord surgery. The retrospective cohort study, however, did not illustrate specific effect of preoperative pressure sores on patients who underwent spinal cord operation, making the association inconclusive.

Thus, we performed a 1:1 matched follow-up study to evaluate the association between preoperative pressure sores and infections after spinal cord surgery. A total of 334 participants were recruited and impact of preoperative pressure sores on various postoperative infections of spinal cord in patients was analyzed.

## Results

### Selection and characteristics of participants

**Figure 1:**
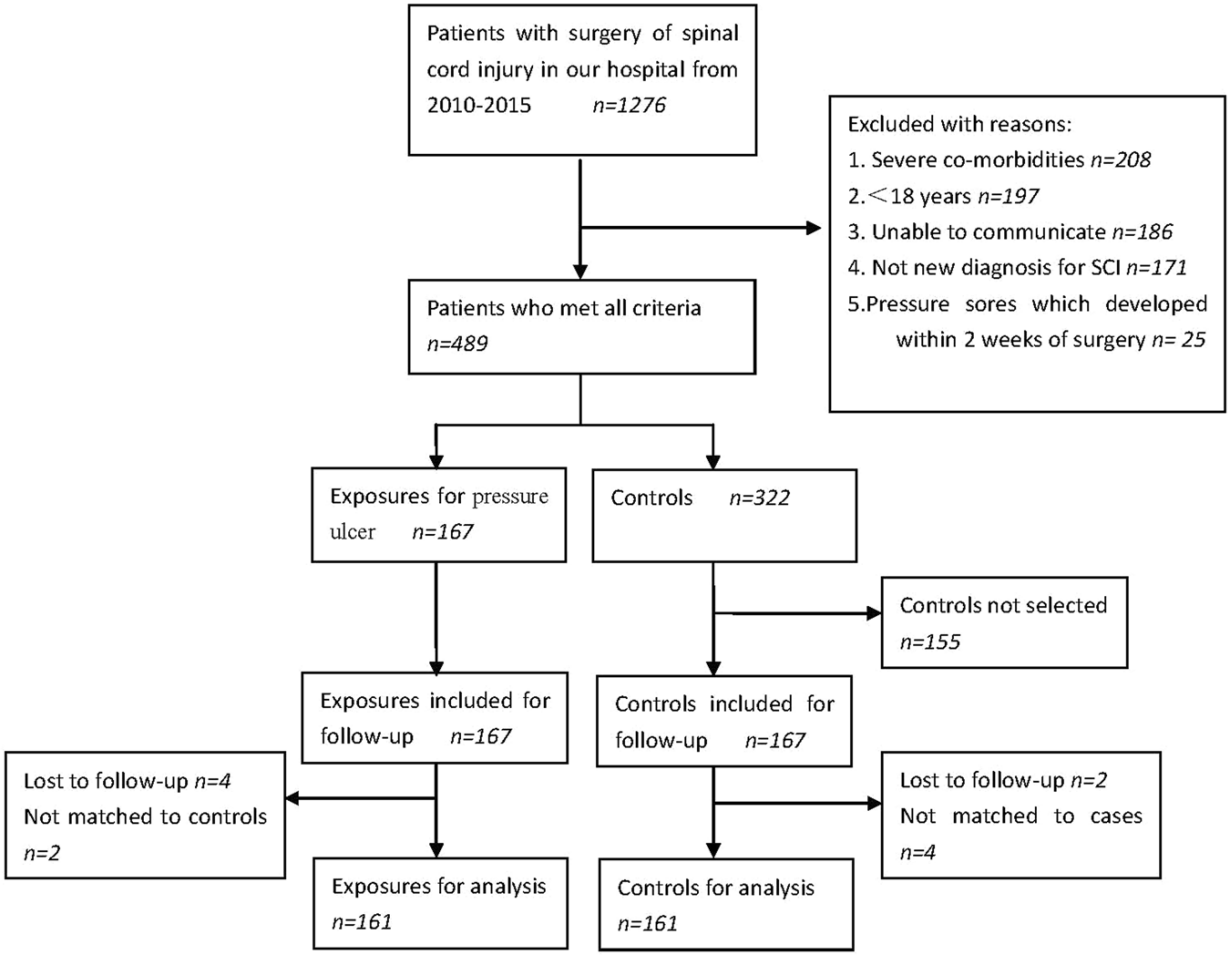
Flowchart exhibiting the selection of eligible individuals in the follow-up study.

A total of 1276 patients who were newly diagnosed SCI within 3 months had operation treatments during January 1, 2010 and December 31, 2015 at the Affiliated Hospital of Guizhou Medical University in China. A total of 334 individuals were recruited, among which 167 patients with decubitus ulcers were the exposures and 167 patients free to decubitus ulcers were the controls (Fig. 1). Data regarding 161 pairs of the exposures and the controls were used for the final analysis since 4 individuals in case group and 2 in control group were lost to follow-up. Among the eligible participants, the mean age was 53.4 ± 7.8 years. There were 55.3% male participants in both groups. Other main characteristics including SCI level, operation procedure and cause for SCI of eligible participants are summarized in Table 1.

**Table 1:** Characteristics of 322 eligible individuals in the follow-up study.

	PU (n = 161)/N(%)	Without PU (n = 161)/N (%)	P-value
Age/years (mean ± std)	52.6 ± 6.4	54.2 ± 5.9	>0.05
Sex			
*Male*	89 (55.3)	89 (55.3)	>0.05
*Female*	72 (44.7)	72 (44.7)	>0.05
SCI level (AIS at initial admission)			>0.05
*A*	76 (47.2)	79 (49.1))	—
*B*	67 (41.6)	63 (39.1)	—
*C*	18 (11.2)	19 (11.8)	—
Operation procedure			>0.05
*Anterior cervical surgery*	112 (69.7)	103 (64.0)	—
*Posterior cervical surgery*	36 (22.4)	49 (30.4)	—
*Combined cervical surgery*	13 (8.1)	9 (5.6)	—
Cause for SCI			<0.05
*Fall injury*	134 (83.2)	92 (57.1)	—
*Traffic accident*	20 (12.4)	45 (14.3)	—
*Other*	7 (4.3)	44 (27.3)	—

Abbreviations: PU = pressure ulcer; AIS = the American Spinal Injury Association Impairment Scale; SCI = spinal cord injury.

### Potential causes for pressure ulcers and its location

We comprehensively reviewed the potential causes for pressure ulcers in participants. Fracture, as the primary cause for pressure ulcers, was accounting for 84.5% (136/161), in particular hip fracture (97/161). Other potential causes for pressure sores included arthritis, cerebral trauma and etc. (Table 2). As for location of pressure ulcers in human body, there were 60.2% (97/161) reports in sacrococcygeal region, 8.7% (14/161) in heel, 7.4% (12/161) in iliac crest, 6.3%(10/161) in greater trochanter, 4.9% (8/161) in ankle, 3.0%(5/161) in ischial tuberosity and 6.3%(10/161) in other regions.

**Table 2:** potential causes for pressure ulcer and its sites.

Potential causes for PU	N (%)	Sites of PU	N (%)
Fractures	136 (84.5)	Sacrococcygeal region	97 (60.2)
*Hip fractures*	97 (60.2)	Heel	14 (8.7)
*Vertebral fracture*	10 (6.2)	Iliac crest	12 (7.4)
*Other fracture*	19 (18.0)	Greater trochanter	10 (6.3)
Arthritis	12 (7.5)	Ankle	8 (4.9)
Cerebral trauma	8 (5.0)	Ischial tuberosity	5 (3.0)
Other	5 (3.1)	Other regions	10 (6.3)

Abbreviations: PU = pressure ulcer.

### Infections risk in relation to preoperative pressure ulcers

Compared with control group, a 3.4-fold (95%CI, 2.1–5.6) increased risk of any kinds of postoperative infections within 14 days was observed in patients with preoperative pressure ulcers (Table 3). The positive association was also pronounced with regard to the specific kinds of infections. RRs for surgical site infection, pneumonia, urinary tract infection were 2.3 (95%CI, 1.1–4.7), 2.4 (95%CI, 1.1–5.3), and 2.8 (95%CI, 1.1–7.3), respectively. After adjusted for possible confounding risk factors, the RR values remained to be statistically significant. Pressure ulcers were also associated with an elevated risk of 30-day postoperative hospitalization for infection (RR, 2.6, 95%CI, 1.1–6.0). However, such positive effects disappeared in septicemia or deep vein infection.

**Table 3:** Risk of infections after surgery of spinal cord in relation to preoperative pressure ulcer.

	PU/N (%)	Without PU/N(%)	RR (95%CI)	Adjusted RR (95%CI)
14-day postoperative infections				
*Surgical incision infection*	25 (15.5)	12 (7.4)	2.3 (1.1, 4.7)	2.2 (1.1, 4.6)
*Pneumonia*	21 (13.0)	10 (6.2)	2.4 (1.1, 5.3)	2.2 (1.4, 3.1)
*Urinary tract infection*	16 (9.9)	6 (3.7)	2.8 (1.1, 7.3)	2.8 (1.2, 5.6)
*Septicemia*	5 (6.2)	1 (0.6)	5.1 (0.6, 44.4)	4.3 (0.8, 35.2)
*Deep wound infection*	2 (1.2)	0 (0)	—	—
*Any of the above*	69 (42.9)	29 (19.2)	3.4 (2.1, 5.6)	3.3 (2.0, 4.2)
30-day postoperative hospitalization for infection	19 (11.8)	8 (5.0)	2.6 (1.1, 6.0)	2.7 (1.2, 5.7)

Abbreviations: PU = pressure ulcer; RR = risk ratio.

### Infections risk in relation to different stages of preoperative pressure ulcers

In terms of various severity degrees of pressure sores, associations between pressure ulcer in either stage III or stage IV and infections post spinal cord surgery were statistically significant but non-significant in stage I or stage II. The highest RR was observed in pressure ulcers of stage IV. In respect to pressure sores of stage IV, RR for 14-day postoperative infection was 4.2 (95%CI, 2.3–6.7) and RR for 30-day postoperative hospitalization for infection was 3.2 (95%CI, 2.0–6.4). The associations were also pronounced after adjusting for the aforementioned specific parameters (Table 4).

**Table 4:** Risk of infections after surgery of spinal cord in relation to different degrees of preoperative pressure ulcer.

	14-day postoperative infections		30-day postoperative hospitalization for infection	
	RR (95%CI)	Adjusted RR (95%CI)	RR (95%CI)	Adjusted RR (95%CI)
PU degree				
I	1.2 (0.9, 2.5)	1.3 (0.9, 2.3)	1.2 (0.9, 3.8)	1.2 (0.9, 4.2)
II	1.9 (1.1, 3.6)	1.7 (1.2, 3.2)	1.3 (1.0, 4.6)	1.3 (0.9, 4.7)
III	3.5 (1.9, 5.8)	3.4 (2.3, 5.5)	2.1 (1.5, 4.6)	2.0 (1.2, 5.0)
IV	4.2 (2.3, 6.7)	4.2 (2.3, 6.7)	3.2 (2.0, 6.1)	3.1 (2.2, 6.4)

Abbreviations: PU = pressure ulcer; RR = risk ratio.

## Discussion

To our best understanding, this is the first study suggesting an elevated infection risk after spinal cord surgery in patients with preoperative pressure ulcers in mainland China. Compared to controls, patients with preoperative pressure sores had a 3.4-fold increased risk of postoperative infections within 14 days and a 2.6-fold increased risk of 30-day postoperative hospitalization for infection. The higher risk of postoperative infections in pressure sore of stage III to IV was identified. The current study suggests a warning to give medical treatment for patients with pressure sores before spinal cord surgery, in particular those with pressure sore of stage III to IV. What is more, it should pay attention to make measures to control postoperative infections.

Several plausible mechanisms might be accounting for increasing risk of infections post spinal cord infections in relation to prior pressure sore exposures. To start with, pressure sores were perceived to induce impairment of skin protection function by destroying integrity of erythematous skin and prompting reproduction and growth of pathogenic bacteria^11^. Several species of pathogenic bacteria including *Staphylococcus, Pseudomonas, Peptoniphilus, Enterobacter, Stenotrophomonas, Finegoldia*, and *Serratia* were detected in collected samples from pressure sores^12^. Pressure ulcers might introduce possible resources and entrances of pathogens to human body, which was possible to result in local infections in human body. Secondly, chronic pressure ulcers were supposed to persist chronic inflammation, which might lead to stimulation of cytokines and inflammation factors^12–14^. An expansive literature suggest that long-term and excessive consumption of cytokines and inflammation factors might be accounting for immune suppression, and in turns led to a weaken ability to prevent invasions and attacks of pathogens^15,16^. Thirdly, immobilization, as a primary risk factor for the occurrence and development of pressure sores^17,18^, was considered to be associated with pneumonia. Immobilization might have destructive effects on removing function of bronchial secretions, which was conductive to reproduction of pathogen and help to result in pneumonia. However, mechanisms about association between preoperative pressure sores and infections post spinal cord surgery are still not conclusive.

This study demonstrates an increased risk of several specific infections (surgical incision infection, pneumonia, urinary tract infection) within 14-day after spinal cord operation in patient with pressure sores preoperatively. Our finding is in line with a retrospective cohort study which showed an increased risk of pneumonia and urinary tract infections after several major operative treatments including skin, breast, spinal cord surgery and etc. in relation to preoperative pressure sores^10^. However, pressure ulcer was neither associated with septicemia nor deep vein infection. What the aforementioned hints that pressure sore might be more easily introduce superficial infections to human body. The possible mechanisms might be involved in migration of pathogen from pressure ulcer to superficial areas.

Patients with different stages of pressure ulcer showed various risk of infections post spinal cord operation. Our finding indicates an elevated risk of either 14-day postoperative infections or 30-day postoperative hospitalization for infection in patient with preoperative pressure ulcers of stage III to IV, however, the elevated risk was towards to null in pressure ulcer of stage I to II. Based on the integrity of erythematous skin and the involved depth of wound, pressure sore of stage I present with intact epidermis, while stage II to IV with skin defect at different levels^19^. Among the four stages of pressure ulcer, stage IV has the highest risk of postoperative infections (Table 4). What the aforementioned suggest a possible dose-response relationship between degree stages of postoperative pressure sores and risk of infections after spinal cord operation. Previous studies showed that there were open wounds, endothelial cell dysfunction, abscess and cellulitis in pressure sores from stage II to IV, which made colony, invasion and infection of bacteria more easily^20,21^.

Even though our analysis was on basis of a follow-up study and results were relatively robust, there were some potential limitations which should be discussed. First of all, specific species of bacteria in wounds of pressure sores or postoperative infections were not detected. Whether postoperative infection resources came from pressure sores was not verified. What is more, the impact of pressure sores occurrence post spinal cord surgery on postoperative infections was not evaluated. According to statistics, morbidity of pressure sore was high among patients post surgical procedures of spinal cord^22^, while occurrences of pressure sores were inconsistent in disease course. Owing to the limited participants, such analysis was failed to conduct. In addition, the study might not avoid affecting by confounding bias. Some factors such as lifestyle, family support, nutrition and etc. have involvement in development of infections^23^. Although we have adjusted several factors for analysis, potential effect of other confounders might remain.

## Conclusions

This follow-up study based on 167 pair of cases and controls demonstrated a positive association between preoperative pressure sores and infections after spinal cord surgery. Preoperative pressure sores might be a risk factor for infections after spinal cord surgery, especially in patients with pressure sore in stage III to IV. In consideration of limited number of participants in this study, additional studies, in particular perspective cohort studies based on population are needed to confirm the impact of preoperative pressure sores on postoperative infections.

## Methods

### Study design

A 1:1 matched follow-up study was conducted. Patients with pressure ulcers were consecutively recruited as the exposures according to inclusion criteria as follow: (1) ≥18 years and capable of communicating and providing information; (2) new diagnosis for SCI within 3 months; (3) diagnosis for pressure sores at least two weeks before SCI; (4) without severe medical co-morbidities including cancer, cardiovascular disease, mental disorder or severe infection over the last two months. For each exposures, control was selected among the clinic patients who were free of pressure ulcers and matched by sex, age (±3 years) with the specific case. Both case and control group underwent operation of spinal cord. The protocol was performed in accordance with the research ethics principles of the Committee of Guizhou Medical University. Written informed consents of this study were obtained from all participants.

### Exposures

Patients with SCI were identified according to the ninth revision of the International Classification Diseases (ICD) for SCI (ICD-9-CM 806). Afterwards, spinal cord impairments were quantified into four levels (A, B, C, D) by the American Spinal Injury Association Impairment Scale (AIS)^24^. Patients received various surgical procedures including anterior cervical surgery, posterior cervical surgery and combined cervical surgery according to their injured sites and severity. Cases with pressure ulcers were initially classified on basis of ICD-9-CM 707.0 and severity levels of pressure sores were graded into four stages (stage I to IV) using the National Pressure Ulcer Advisory Panel^18^. Characteristic information of participants was collected with a designed questionnaire. Causes of pressure sores in the exposure group were comprehensively studied.

### Outcomes

Postoperative infections within 14 days, including surgical site infection (ICD-9-CM E878), pneumonia (ICD-9-CM 480-486), urinary tract infection (ICD-9-CM 599.0), deep vein infection (ICD-9-CM 958.3), septicemia (ICD-9-CM 038, 998.5), were regarded as primary outcomes. Secondary outcome was 30-day postoperative hospitalization for infection.

### Statistical analysis

Risk ratios (RRs) and their corresponding 95% confidence intervals (CIs) for associations between pressure ulcers and risk of infections after spinal cord operation were calculated by unconditional logistic regression analysis after adjusting for sex, age. Additionally, Subgroup analysis was conducted, which stratified by pressure ulcer degree (stage I to IV). All statistical analysis was conducted using SPSS 13.0 software (SPSS Inc., Chicago, IL, USA). Statistical tests in this study were 2-sided and a P value < 0.05 for statistically significance was used.
